# Preparation of magnetic nanoparticle integrated nanostructured lipid carriers for controlled delivery of ascorbyl palmitate

**DOI:** 10.1016/j.mex.2020.101147

**Published:** 2020-11-17

**Authors:** Gokce Dicle Kalaycioglu

**Affiliations:** Chemical Engineering Department, Hacettepe University, Turkey

**Keywords:** Biotechnological drugs, Controlled drug delivery, Ascorbic acid, Magnetic nanoparticles, Nanostructured lipid carriers

## Abstract

Most cancer treatments can cause vital side effects on healthy tissues. Ascorbic acid (AA) is a water-soluble antioxidant molecule and possesses a variety of functions such as prevention of tumor proliferation and treatment of cancer. However, AA, is very sensitive to air, heat and light. Its high hydrophilicity also makes the controlled delivery difficult. To overcome these problems, AA can be chemically-modified and made more hydrophobic by the esterification. Palmitic acid is one of the most common long-chain fatty acids that can be used for this purpose. It is known that Ascorbyl palmitate (AP) which is a lipopihilic derivative of AA, can inhibit cell proliferation and DNA synthesis in many types of cancer. Although AP has higher stability, its bioavailability and therapeutic effect is low due to its lipophilicity and low release capacity.

• In this study, nanostructured lipid carriers (NLC) which are colloidal nanoparticles with high biocompatibility, low crystallinity and high hydrophobic-drug encapsulation capacity was prepared to increase the bioavailability of AP.
• To provide triggered drug release via hyperthermia, magnetic nanoparticles (MNps) were integrated into the NLCs besides AP.
• The synthesis of biocompatible NLCs with controlled and triggered release ability, is successfully completed and controlled release of AP as an antitumor agent is achieved.

## Graphical abstract

**Figure:**
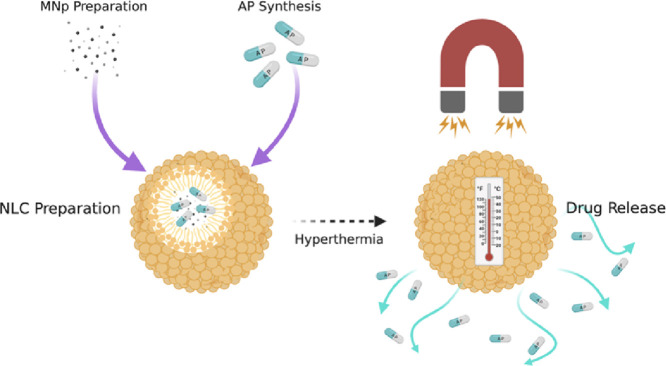

**Specifications table**

**Table:** 

Subject Area:	Materials Science
More specific subject area:	*Biotechnological drug delivery systems*
Method name:	*Preparation of controlled drug delivery systems*
Name and reference of original method:	*AP Synthesis*[1] *MNp Preparation*[2], [3], [4]
Resource availability:	*–*

## Method details

### Overview

Ascorbic acid (AA), also known as vitamin C, is an important water-soluble molecule which is necessary for many important biosynthesis such as carnitine, collagen and neurotransmitter molecules in human body [5]. Being a strong antioxidant, AA, which is known to help prevent many diseases caused by oxidative regeneration, has also been discovered in recent studies to have antitumor properties when applied in high doses [6,7]. On the other hand, some other studies showed that overdose AA can increase the risk of cancer instead of preventing it. Looking at the studies in recent years, it is proved that high dose of AA exhibits antitumor effect if administrated intravenously or intraperitoneally [8,9]. The problems encountered at this stage are that the administration of high doses of active agent to the patient by these means is not easily tolerable and the desired therapeutic level is difficult to reach in the tumor area. Therefore, administration of AA in a suitable nanocarrier can greatly increase the therapeutic effect of AA. In addition, the problem of sensitivity to light, heat and air and being easily degradable due to these factors can be overcome by chemically modifying AA with a long-chained fatty acid via an esterification reaction [10]. Also, since AA has high solubility in water, there is a high risk of burst release through the carrier. This situation is in contrast to the logic of administering AA in a nanocarrier. Making AA more hydrophobic by chemical modification will allow it to diffuse more slowly through the carrier, resulting in a more sustainable release. Palmitic acid is one of the most common long-chain fatty acids that can be used for this purpose [11]. It is known that ascorbyl palmitate (AP) which is a lipophilic derivative of AA, can inhibit cell proliferation and DNA synthesis in many types of cancer. Although AP has higher stability, its bioavailability and therapeutic effect is low due to its lipophilicity and low release capacity [11].

Nanostructured lipid carriers (NLCs), which are developed as a new generation lipid particle, are composed of a mixture of solid and liquid lipids [12], [13], [14]. Incorporation of liquid lipids into the structure causes a melting point depression compared with the original solid lipid, but the matrix is still solid at body temperature. Compared to solid lipid nanoparticles (SLN), the less perfect crystalline structure of NLCs leave enough space to accommodate drug molecules which facilitate an improved loading capacity for various active compounds and stable drug incorporation during storage [15]. NLCs possess a number of advantageous features in terms of drug delivery. Besides their small size and biocompatible structure, NLCs are able to enhance the solubility of hydrophobic drugs and the chemical stability of compounds sensitive to light, oxidation and hydrolysis [16].

Moreover, MNPs have been used in many biomedical applications, such as magnetic hyperthermia, magnetic resonance imaging and drug delivery systems [17], [18], [19]. Magnetic hyperthermia (MH) is based on induction heating of MNps under application of an alternating magnetic field and the temperature increase in body tissues leading to cellular structure change. In high temperature, such as 40–43 °C, cancerous cells would be damaged but healthy cells survive. Basically, MH is focused on the destruction of tumors by heat, which is obtained from magnetic nanoparticles under the application of an alternating magnetic field (AMF) [17], [18], [19]. In addition to the use of MNPs individually, their integration into a drug-loaded nanocarrier and the application of local HP allows a combined therapy with a dual effect, while causing damage to the tumor cells locally and promoting the diffusion of the drug through the carrier. In this context, the modification of AA, as an antitumor agent, with PA and the administration of AP with MH by loading into MNp integrated NLCs (MNLCs), both providing locally high dosage AA application and local damaging of tumor cells can be considered as a promising and advantageous method that can be used in combined therapy.

### Materials

Stearic acid, Tween 80, disodium phosphate and monosodium phosphate were purchased from Merck (Darmstadt, Germany). Oleic acid (OA) was supplied from Fisher Chemicals (Loughborough, UK). Iron (III) chloride, sodium hydroxide, palmitic acid (PA), potassium permanganate, sulfuric acid (99%) and ascorbic acid were obtained from Sigma Aldrich (St. Louis, MO, USA). Iron (II) sulfate heptahydrate was purchased from Riedel-de Haën (Hanover, Germany). Nitric acid was supplied from Carlo Erba Reagenti (Milano, Italy). Ethanol, acetone and other reagents were used in analytical grade. Ultra-purified water (UP) was used for chemical synthesis (Millipore Direct-Q3 UV). All materials and reagents were used without further purification.

#### Ascorbyl palmitate (AP) synthesis

In the synthesis of AP, the method that Wen et al. was developed via ultrasonic technology is used [1]. However, in this study, instead of ultrasonic device, as it is a cost-effective application and more suitable for scale-up, traditional mechanical stirring was utilized.

Below are steps involved in synthesis;

1. The reaction of PA and AA in sulfuric acid.
2. Cooling the reaction mixture with crushed ice.
3. Extraction with ethyl ether via separating funnel, solvent removing and filtering.

It is recommended to use lab-coat, gloves and eye goggles as personal protective equipment while performing the synthesis of AP. The synthesis and handling of all chemicals should be performed under the chemical hood.

The details of each step involved in AP synthesis is provided below.

1. ***The reaction of PA and AA in sulfuric acid***

It is known that the ratio of PA to AA to reach optimum efficiency (80–85%) is 1.35: 1.0. In the synthesis, 1.84 mmol AA, 2.48 mmol PA and 5 ml sulfuric acid (99%) per 1 g AA were used.

**Fig. 1:**
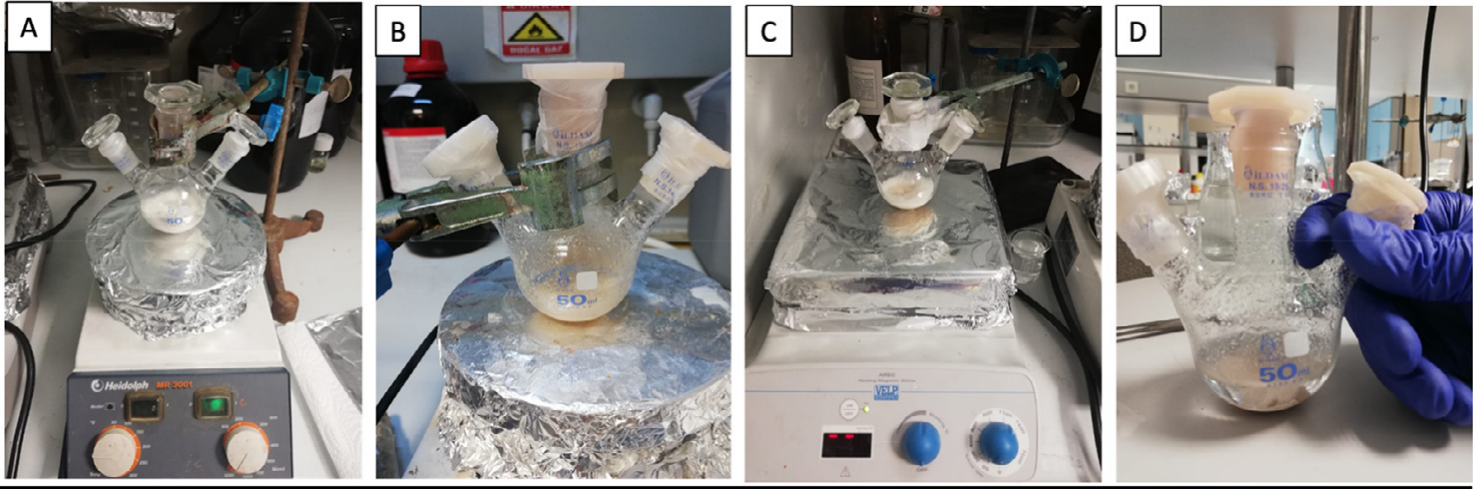
**A)** Dissolving PA in sulfuric acid, **B)** addition of AA, **C)** PA and AA reaction in sulfuric acid, and **D)** light beige-whitish color product in sulfuric acid.

Firstly, 0.637 g PA and 1.62 ml sulfuric acid were put into the reaction vessel and the PA was dissolved at 35 °C. This process took approximately 100 min under stirring at 500 rpm (Fig. 1.A). Subsequently, 0.324 g of AA was added to the round-bottomed glass reaction flask and the reaction continued for 40 h. At the end of the reaction, the solid product in the reaction flask turned into a light beige-whitish color.

2. ***Cooling the reaction mixture with crushed ice***

**Fig. 2:**
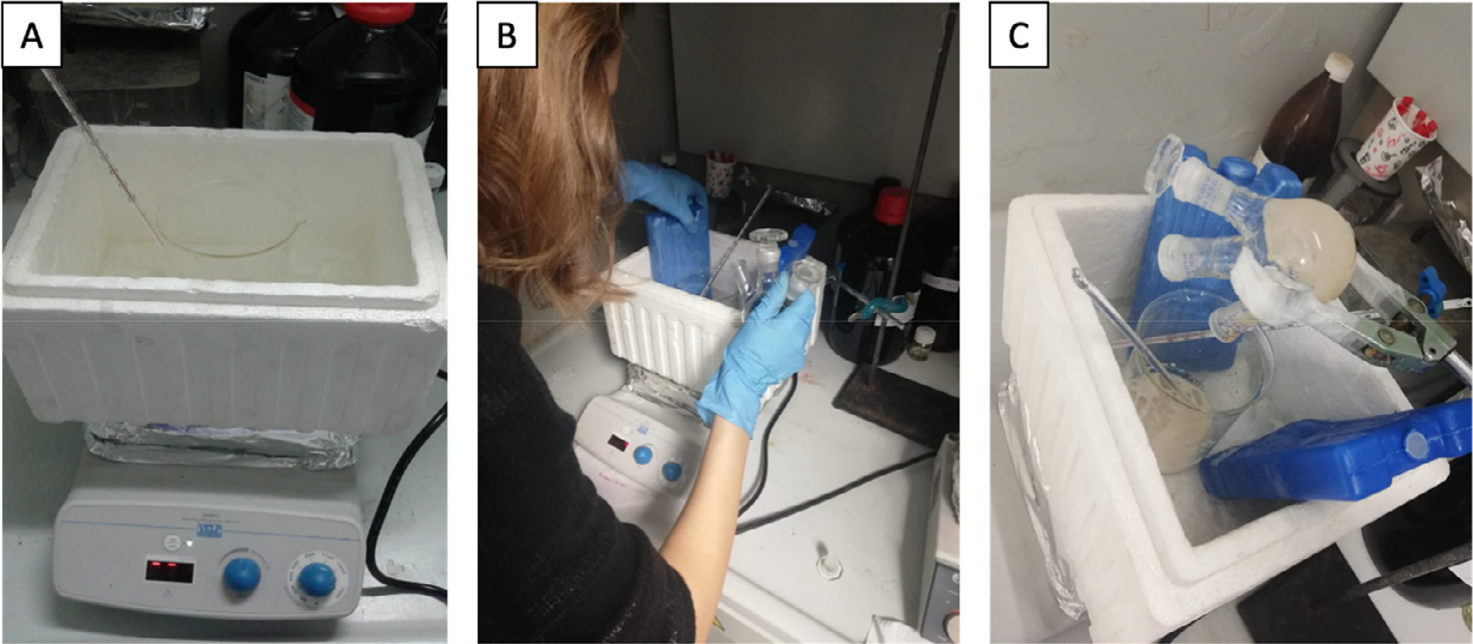
**A)** Crushed ice in an outer ice bath, **B)** addition of reaction product into the crushed ice and, **C)** vigorous stirring of ice and the reaction product.

Just before the termination of the reaction after 40 h, 14.72 g of crushed ice was prepared in an ice bath (Fig. 2A). After the reaction was terminated, the material in the reaction flask was quickly poured into the beaker with crushed ice (Fig. 2B). It was ensured that the ice and the reaction product were mixed homogeneously with a vigorous stirring, taking care not to increase the temperature above 5 °C (Fig. 2C).

3. ***Extraction with ethyl ether via separating funnel***

**Fig. 3:**
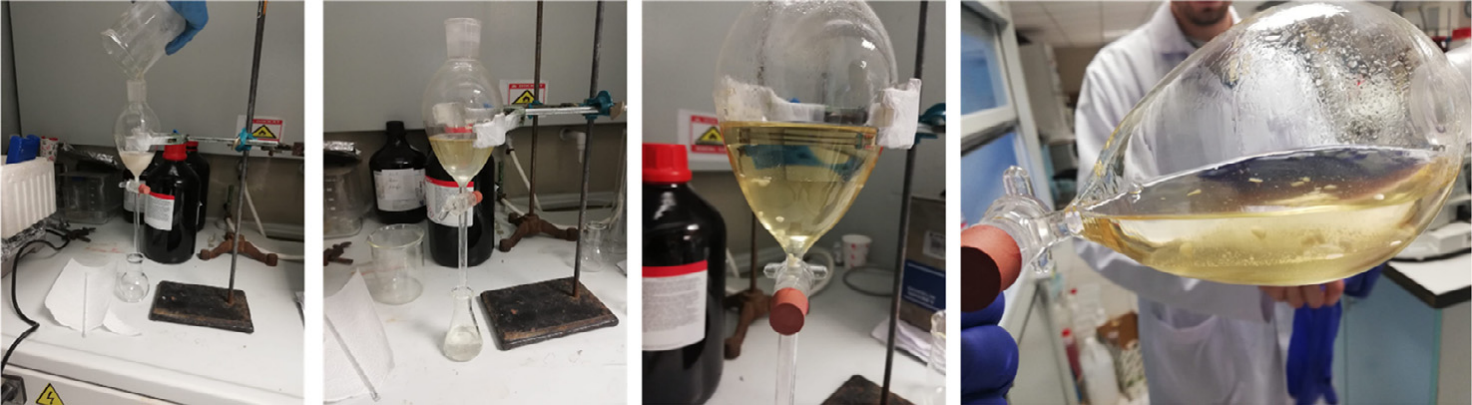
Extraction with ethyl ether in separating funnel.

**Fig. 4:**
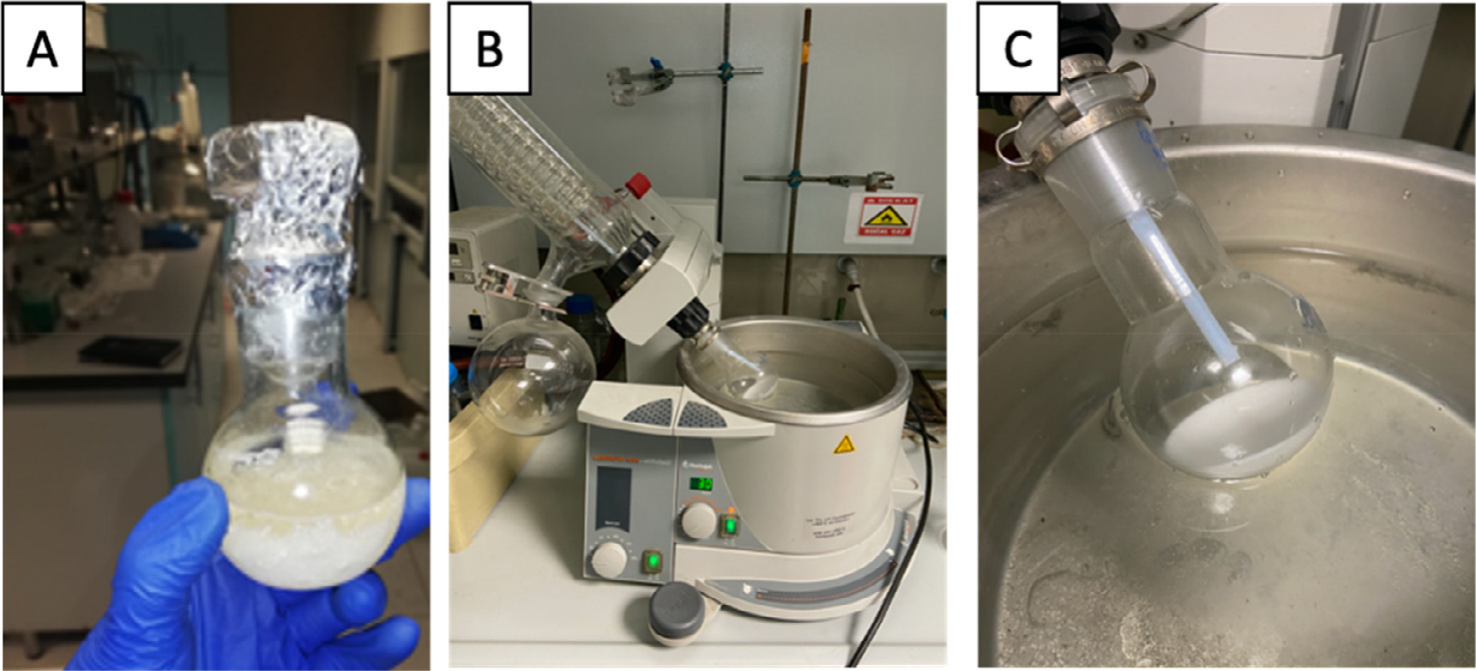
**A)** Washing with brine solution and **B), C)** solvent evaporation with rotary evaporator.

**Fig. 5:**
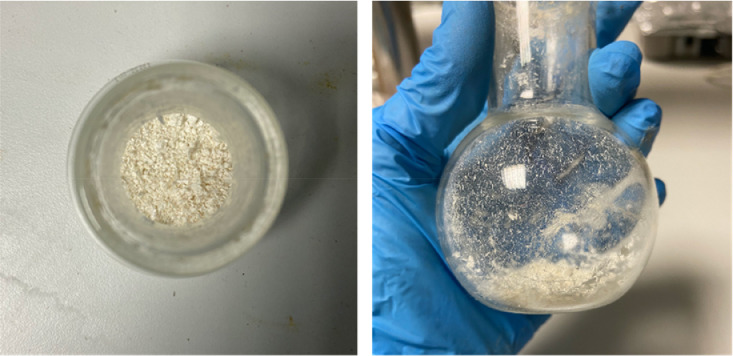
Synthesized and purified AP.

The cold mixture obtained was taken into the separating funnel with ~50 ml ethyl ether and extracted 3 times (Fig. 3). The ether phase was then washed with half-saturated brine and filtered (Fig. 4). Then, the solvent was evaporated using a rotary evaporator with the water bath set at 30 °C, and a white solid product was obtained (Fig. 5).

As the last step, the white solid obtained was washed 3 times with 10 ml of petroleum ether. After each ether addition, the reaction flask was kept at 4 °C for 10 min, allowing the solid to precipitate completely. After 3 washes, the product was dried in a desiccator at 25 °C and obtained in a pure form (Fig. 5).

After the synthesis the yield of reaction was calculated as ~85% [1].

**Fig. 6:**
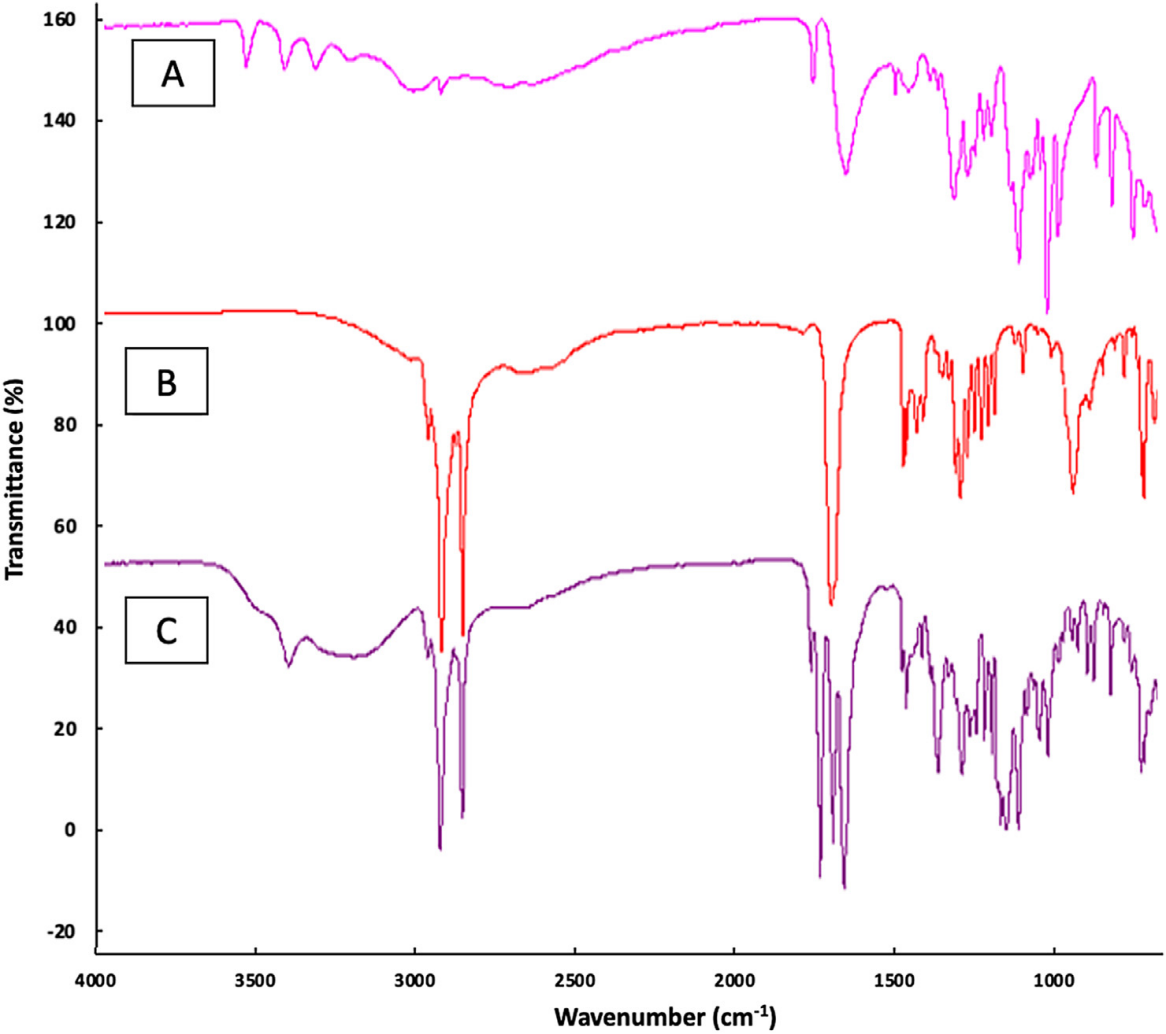
FTIR analysis of **A)** AA, **B)** PA and **C)** AP.

FTIR (Fourier Transformed Infrared) analysis was used for the quantitative determination of the formation of AP (Nicolet 6700 Thermo Scientific). The scanning was carried out on wavenumber 400–4000 cm^−1^. According to structure of the compounds, C=C, C=O, C—H, C—C, C—O–C, C—O–H, C—C(=O)–O functional groups were expected to show peaks in respective regions. As it is seen in Fig. 6C, OH group of AP showed a peak at ~3405 cm^−1^, and –C—H stretching vibration in –CH_2_ at ~3001 and 2915 cm^−1^. In addition, it is seen that C=O of ester moiety has its peak at ~1752 cm^−1^. Moreover, the peak of C=C stretching vibration at ~1652 cm^−1^, C—O–C stretching vibrations in the region of 1000–1400 cm^−1^ and the PA linkage is at ~720 cm^−1^ [20]. As a result, FTIR analysis showed that the reaction was carried out as it should be and the synthesis of AP is achieved successfully.

#### AP-loaded MNp-NLC preparation

In order to prepare AP-loaded MNp-NLCs (AP-MNLCs), first of all MNps must be prepared. Co-precipitation method, which is a well-known method in the literature, has been used for the synthesis of MNps [4]. Since OA will be used as the liquid lipid in the NLC preparation, to increase the encapsulation efficiency of MNPs into NLCs, MNPs were also prepared as oleic acid coated.

**Fig. 7:**
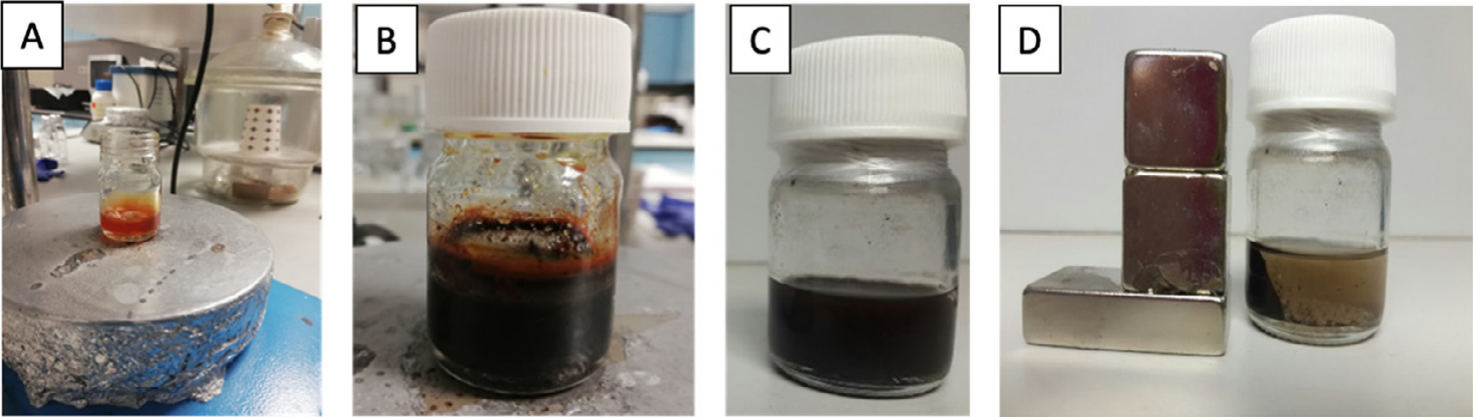
**A)** Fe^2+^/Fe^3+^solution at pH=10, **B)** magnetite dispersion at pH=5, **C)** and **D)** purified MNp dispersion.

First, two solutions were prepared using 0.18 g FeSO_4_·7H_2_O in 1.75 ml up and 0.34 g FeCl_3_·6H_2_O in 2 ml up, in two vials. These two solutions were then combined in a single vial. NaOH solution was prepared with 0.8 g NaOH in 20 mL water and was poured to the Fe^2+^/Fe^3+^ solution until pH reaches to 10 under mechanical stirring (Fig. 7A). After this step, 80 µl oleic acid was added to solution and the emulsion was aged, under mechanical stirring at room temperature for 1 h. To convert iron hydroxides into magnetite, the temperature was heated up to 95 °C (2 °C/min) and then cooled down to room temperature. Solution pH was measured and adjusted to pH 5 with HNO_3_ solution (35% HNO_3_) (Fig. 7B). The precipitated particles were washed with UP for four times to remove the salts and then washed with acetone to remove water and excess OA (Fig. 7C and D).

**Fig. 8:**
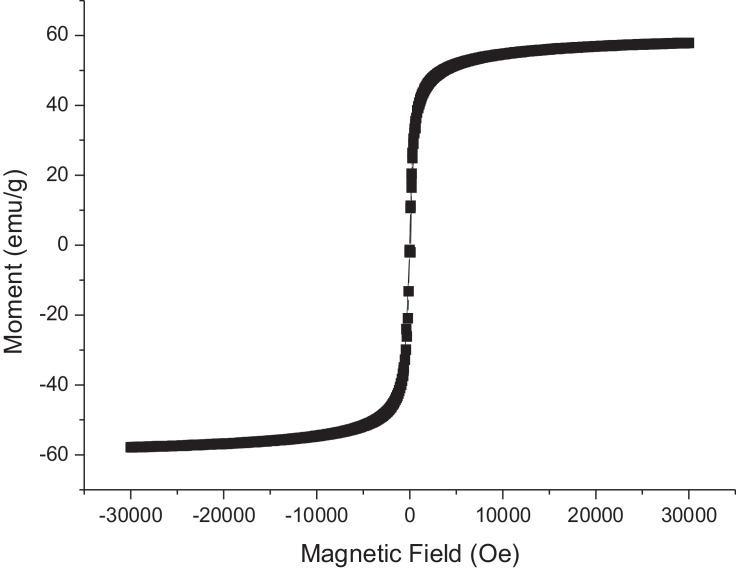
VSM result of OA coated MNPs.

The diameter of the MNps was measured by using Dynamic Light scattering (DLS) as 16±2 (Table 1) nm and the magnetization properties of MNps were investigated with a vibrating sample magnetometer (VSM, Quantum Design) at 25 °C in the field H range of ± 40,000 Oe. As it is seen in Fig. 8, it is observed that the saturation magnetization value of OA coated MNps as ~60 emu/g and the particles showed no coercivity which indicates their superparamagnetic property [21].

**Table 1:** Diameters of the nanoparticles measured by using DLS (*n* = 3) (±SD).

Particle	Diameter (nm)
NLC	57±4
MNp	16±2
AP-MNLC	144±11

After the preparation of MNps, the process of preparing NLCs was started. For this, melt-emulsification method is used, which is a fast and inexpensive method that does not leave toxic residues and does not require the use of expensive or complicated equipment [22].

**Fig. 9:**
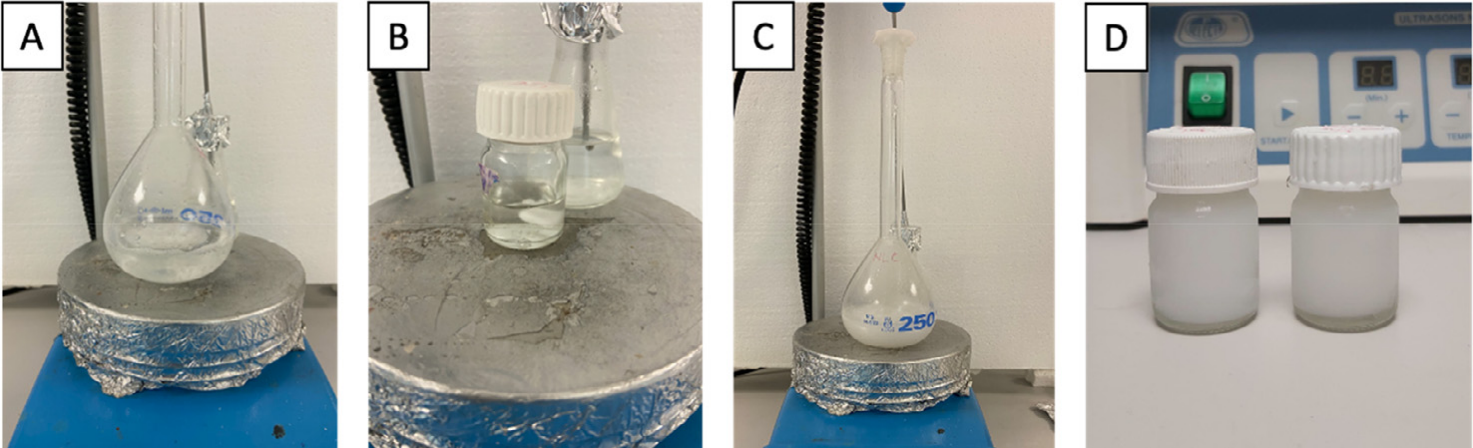
**A)** Aqueous phase, **B)** lipid phase, **C)** aqueous and lipid phase mixture, and **D)** NLC dispersion.

Stearic acid was used as solid lipid, oleic acid as liquid lipid and Tween 80 as emulsifier for NLC preparation. The aqueous phase (Fig. 9A) and the lipid phase (Fig. 9B) were prepared in separate vials and heated up to 75 °C. First, 0.0665 g stearic acid and 31.8 µl OA were added to lipid phase vial. Then 3 ml acetone and 3 ml ethanol were poured onto the lipid mixture. If AP and MNp loaded NLCs (AP-MNLCs) were to be prepared, they were added after ethanol was poured. When the temperature of both solutions reached to 75 °C, lipid phase was injected into the aqueous phase in one move by using glass syringes (Fig. 9C). Then the mixture was stirred at 1000 rpm for 30 min and rapidly cooled to 0 °C to solidify the NLCs (Fig. 9D).

**Fig. 10:**
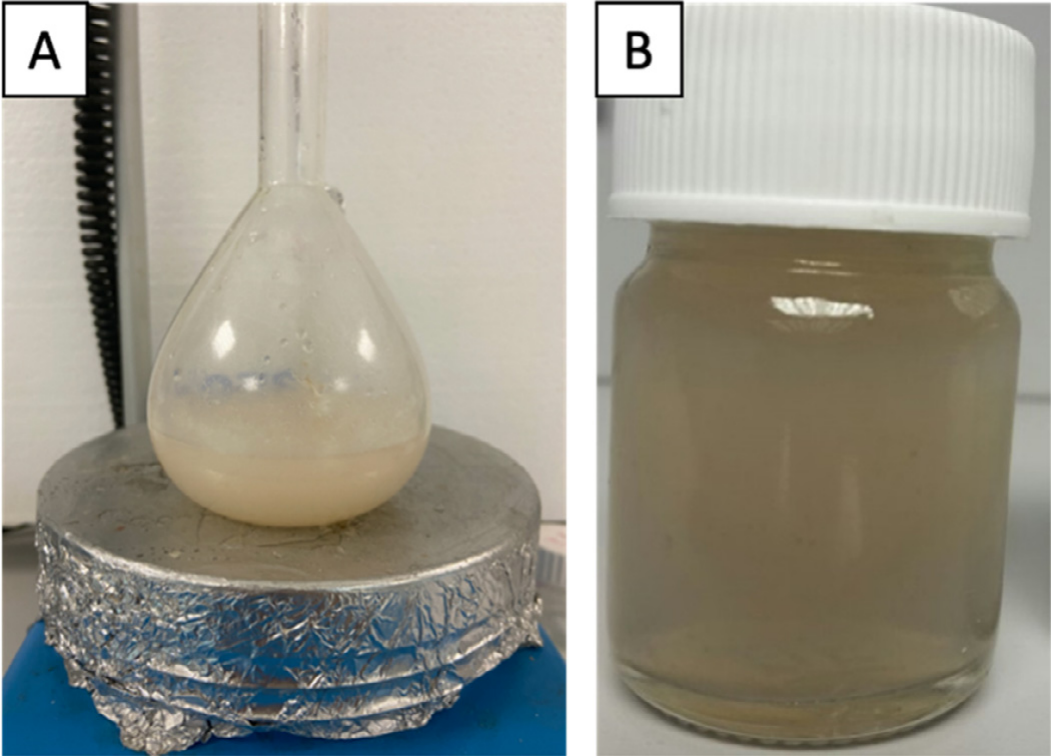
**A)** Aqueous and lipid phase mixture and **B)** AP-MNLC dispersion.

To prepare AP-MNLCs, 0.076 g AP and 0.021 mg MNp were carefully weighed and added after ethanol was added to the lipid phase. Then, NLC preparation continued normally and the prepared lipid phase was added to the aqueous phase (Fig. 10A). At last, the temperature was rapidly reduced to 0 °C and AP-MNLCs were formed (Fig. 10B).

**Fig. 11:**
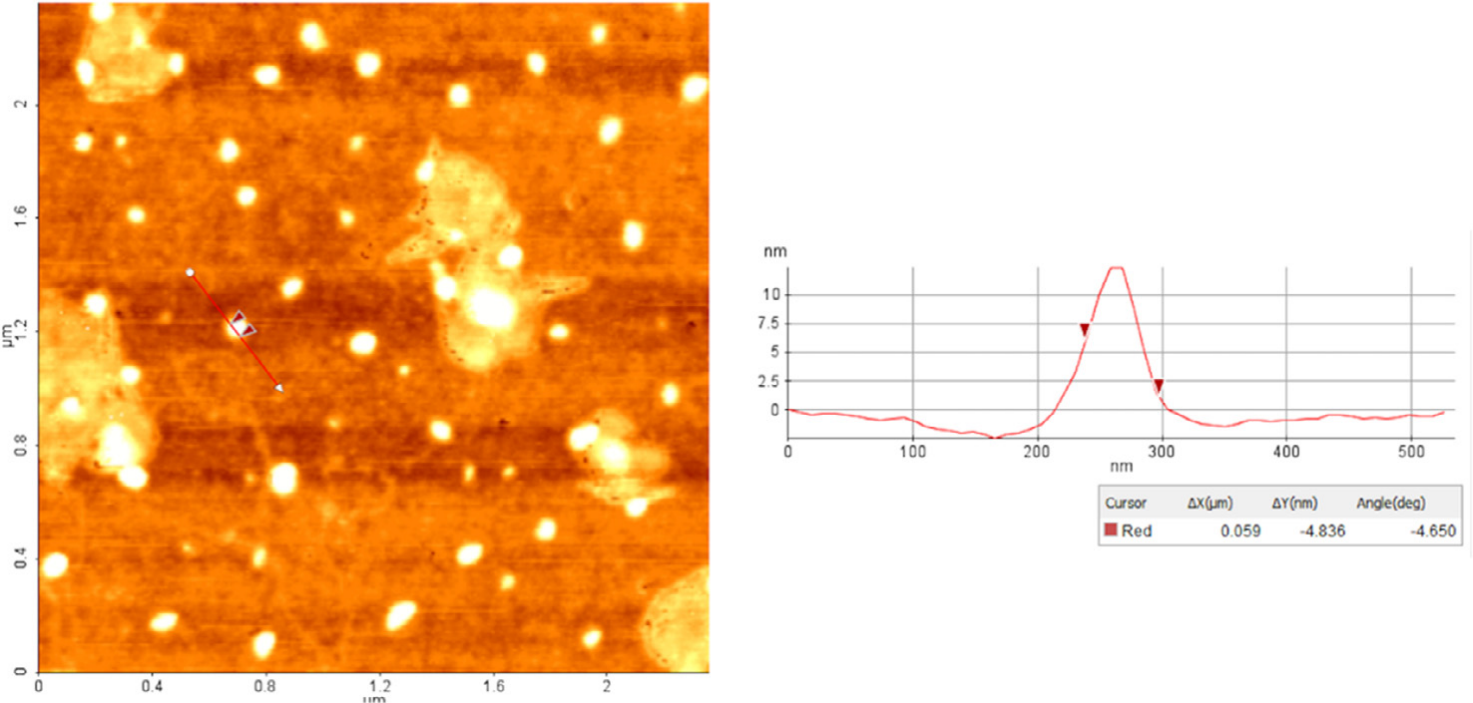
AFM image of NLCs in “topography” mode.

The diameters of the NLCs were measured using AFM and DLS. As can be seen in the AFM image given in Fig. 11, monodispers and spherical particles were successfully prepared. As given in Table 1, the diameters of the particles have been determined to be suitable for use in biomedical applications.

When the drug encapsulation efficiency values were examined, it was seen that AP encapsulation was quite high even in the presence of MNp (Table 2).

**Table 2:** Encapsulation efficiency% values of NLCs.

Particle	Encapsulation Efficiency%
AA-NLC	78±2
AP-NLC	97±1
AP-MNLC	88±3

**Fig. 12:**
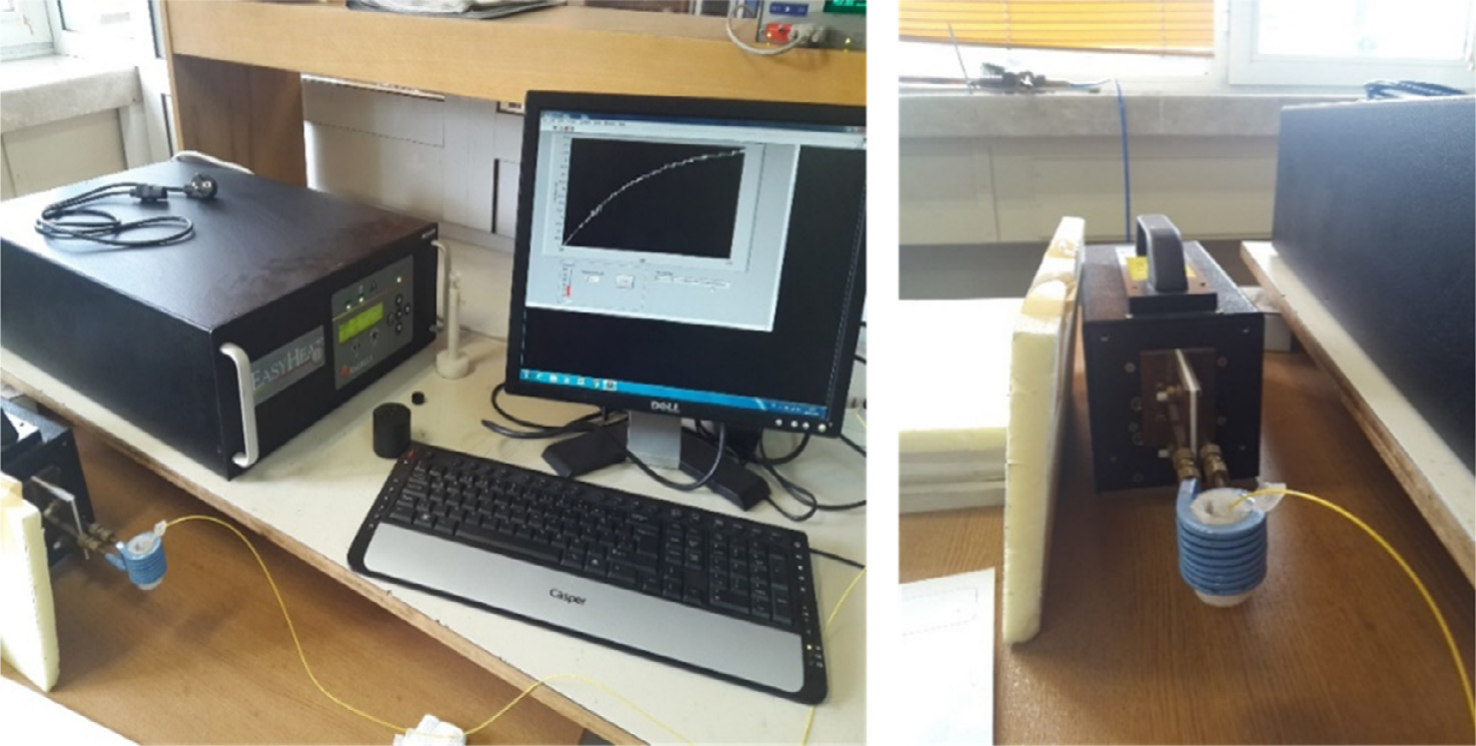
Hyperthermia heat induction system with an external AC magnetic field generator with 8 turns of a copper coil.

The hyperthermia studies were carried out by using the Ambrell Easy Heat induction heater and the measurements were performed via a custom-made computer-controlled system. The induction heater has an external AC magnetic field generator with 8 turns of a copper coil (Fig. 12). The sample was placed inside the coil which has an outer diameter of 3.2 cm and an inner diameter of 2.5 cm. This system can work with a frequency range between 325 kHz to 302 kHz and generate a maximum 150 A of current value. In a typical experiment, NLC dispersions were placed into the center of magnetic field generator and increment of temperature was measured by fiber optic temperature sensor. All samples were placed in 1.5 mL centrifuge tube and surrounded by insulating Styrofoam. In order to mimic real physiological conditions, hyperthermia experiments were performed at 36 °C with a constant 47.7 kA/m magnetic field.

### Method validation

The drug release experiments were conducted in a phosphate buffer solution (PBS) at pH=7.4 and at 37±1 °C. 12 ml of NLC dispersion was poured into a dialysis bag (MW cut off 14.000) and placed into 80 ml release medium under magnetic stirring (50 rpm). At suitable time intervals, 500 µL aliquots of samples from release media were withdrawn and the amount of drug released was analyzed with UV–vis spectroscopy (Thermo Scientific™, GENESYS 10S) and the permanganometric method [23]. In this method, the sample that contains AA or AP was incubated in1 ml of 0.1 mg/ml KMnO_4_ solution (prepared using 5 M H_2_SO_4_). After 5 min aging at room temperature, it is observed the transformation of purplish-colored potassium permanganate to colorless potassium ascorbate through the Reaction (1).

(1) $$\text{KMn}O_{4}+HC_{6}H_{7}O_{6}\;\to KC_{6}H_{7}O_{6}+\;H^{+}{\text{MnO}}_{4}^{-}$$

The residual solution was then analyzed using UV–vis spectroscopy at 525 nm wavelength, the released amount of drug was calculated via pre-formed calibration graph and the drug release (%) values were calculated by using Eq. (2) [23].

(2) $$Drug\;Release\;\%=\;\frac{Amount\;of\;released\;drug}{Total\;amount\;of\;encapsulated\;drug}\times 100$$

**Fig. 13:**
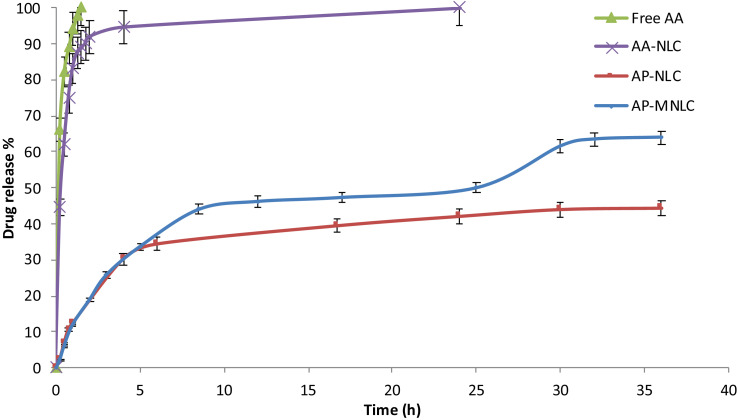
Drug release% values as a function of time for **(A)** free AA, AA-NLC, AP-NLC and AP-MNLC. **(B)** The arrows indicate the time that hyperthermia applied.

When we look at the drug release% values in Fig. 13.A, it is seen that there is not much difference between free AA and AA-NLC in terms of percentage release. Looking at the released value after 1 h, it is seen that free AA is only 11% more than AA-NLC. This is the main reason why AA is chemically modified with palmitic PA and converted to AP. When looking at AP release from AP-NLC, it is observed that the release is slowed down considerably and converted into a controlled manner. While AA-NLC release% value was 83% after 1 h, this value was calculated as 23% for AP-NLC.

Moreover, in Fig. 13.B, it is clearly seen that when hyperthermia is applied to the sample, the release is facilitated and the percentage release amount is considerably increased (orange arrows). For the first application of hyperthermia, the release is increased from ~33% to 44~and, for the second application, from ~50% to ~62%

The results obtained show that the encapsulation of AA into NLC by chemically-modified and converted to AP in the presence of MNp enables drug release to be carried out in a highly controlled, sustainable and at the same time in a triggered manner.

These findings showed that the method presented here is valid as well as promising and advantageous in terms of biomedical applications.

## Declaration of Competing Interest

The authors declare that they have no known competing financial interests or personal relationships that could have appeared to influence the work reported in this paper.
